# Identification of potential CpG sites for oral squamous cell carcinoma diagnosis via integrated analysis of DNA methylation and gene expression

**DOI:** 10.1186/s12957-021-02129-1

**Published:** 2021-01-19

**Authors:** Le Chen, Dong Wang

**Affiliations:** Department of Stomatology, The Second Hospital of Tianjin Medical University, 23 Pingjiang Road Intersection, Hexi District, Tianjin, 300211 China

**Keywords:** OSCC, Diagnosis, DNA methylation, Gene expression, Logistic regression

## Abstract

**Background:**

Oral squamous cell carcinoma (OSCC) accounts for more than 90% of the oral carcinomas and has a high fatality rate. This study aimed to identify potentially diagnostic biomarkers of OSCC through integrated analysis of DNA methylation and gene expression profiles.

**Methods:**

The DNA methylation profiles of OSCC patients from The Cancer Genome Atlas (TCGA) were analyzed to screen patients with CpG island methylator phenotype (CIMP) and investigate the relationship between CIMP and survival probability of OSCC patients. Differential methylation and expression analyses of the paired OSCC tumor and paracancerous samples from TCGA were performed. Logistic regression model was established, and the accuracy of this diagnostic model for OSCC was evaluated in validation sets from Gene Expression Omnibus (GEO).

**Results:**

OSCC patients with CIMP had lower survival probability than those without CIMP. The cg02860732 and cg04342955 were determined as candidate diagnostic methylation sites for OSCC. Logistic regression model was established based on cg02860732 and cg04342955 showed relatively high diagnostic accuracy in OSCC.

**Conclusions:**

A diagnostic model for OSCC was identified based on the methylation sites cg02860732 and cg04342955, which might be favorable for the diagnosis of OSCC.

**Supplementary Information:**

The online version contains supplementary material available at 10.1186/s12957-021-02129-1.

## Introduction

Oral squamous cell carcinoma (OSCC) is a malignant tumor occurring in the oral cavity accounts for more than 90% of the oral carcinomas [1]. As a frequent cancer, there are approximately 500,000 new cases diagnosed with OSCC annually [2]. In spite of the advances in therapeutic strategies including chemotherapy, radiotherapy, and surgery, OSCC still has a relatively high mortality of 48%, remaining to be a severe health burden worldwide [3, 4]. Although biopsy and histopathological exams were gold standard for OSCC diagnosis, a large percentage of OSCC cases are diagnosed at advanced stage, which results in the grave prognosis, it is generally believed that early and accurate diagnosis plays a pivotal role in ameliorating the survival rate and prognosis of OSCC [5]. Genetic molecular mechanism exploration of occurrent and development of OSCC was critical for monitoring disease progression earlier.

An increasing number of studies have been investigating the OSCC-related biomarkers. A previously reported study showed that cytokeratins (CKs) like CK17, which exhibited the most pronounced upregulation among the identified CKs in OSCC samples compared with the normal controls, were a possible diagnostic biomarker in OSCC [6]. Ishiwata et al. found that heat shock factor 1 (HSF1) expression level in nuclear had close association with OSCC development, including the tumor size and histopathologic types, which might act as a potential tool for OSCC diagnosis [7]. Tang et al. indicated that some long non-coding RNAs (lncRNAs) were abnormally expressed in OSCC, presented detectable amounts in saliva, and could be adopted as non-aggressive diagnostic biomarker in OSCC [8]. Except for the aberrant expressions of mRNA and lncRNA, epigenetics has been a promising field in cancer research, including DNA methylation which occurs in the CpG islands near gene transcription start sites [9]. Alteration of DNA methylation is able to affect the gene expression, as well as diverse molecular mechanisms; thus, aberrantly methylated CpG sites are regarded as promising biomarkers in various cancers including OSCC [10, 11]. For example, Foy et al. found that the abnormal methylation of genes *FOXI2*, *AGTR1*, *PENK*, and *LINE1* is a possible indicator of OSCC development [12]. Langevin et al. identified 7 methylation sites which were related to the prognosis of OSCC patients and might be used as non-invasive detection tools in clinical practice [13]. However, study of epigenetics focuses on the mechanism of OSCC progression. To obtain the reliable diagnosis signature, study of differential expression of genes should be analyzed between cancer samples and paracancerous normal samples. After comprehensively analyzing the two expression profiles, the downstream diagnosis could be found more accurately and reasonably based on the methylation molecular mechanism of OSCC.

In this study, the DNA methylation and gene expression profiles of OSCC samples from The Cancer Genome Atlas (TCGA) were comprehensively analyzed to identify potentially diagnostic methylation sites. The diagnostic value of the screened biomarkers was further validated in two validation sets from the Gene Expression Omnibus (GEO) by logistic regression analysis. Our study might provide novel promising signatures for the diagnosis of OSCC.

## Material and methods

### Data source

The gene expression profiles of 75 OSCC samples were obtained from The Cancer Genome Atlas (TCGA, www.cancergenome.nih.gov), including 65 OSCC tumor samples and 5 pairs of tumor and paracancerous samples. The HTSeq-Counts data were downloaded for subsequent analysis.

The DNA methylation data were downloaded from TCGA and Gene Expression Omnibus (GEO, https://www.ncbi.nlm.nih.gov/geo/). The TCGA dataset consisted of the DNA methylation data and clinical information of 75 OSCC samples, including 65 OSCC tumor samples and 5 pairs of tumor and paracancerous samples. The GEO dataset GSE87053 included 11 OSCC tumor samples and 10 normal controls, and GSE123781 included 23 OSCC tumor samples and 18 normal controls. The DNA methylation profiles were all measured by Illumina Human Methylation 450 (HM450) arrays.

### Identification of CpG island methylator phenotype (CIMP)

The methylation sites with standard deviation (SD) > 0.2 in 70 OSCC patients and *β* < 0.05 in 5 paracancerous samples were selected [14, 15]. Based on these methylation sites, the 70 OSCC patients were clustered using K-means method; then, the patients with CIMP were identified according to the methylation level of each cluster.

### Differential methylation and expression analyses

Differential methylation analysis was performed for the 5 pairs of OSCC and paracancerous samples. The methylation sites with missing values or those on the sex chromosomes were firstly removed; then, paired *t* test was used for differential methylation analysis, with |△β| > 0.2 and *p* < 0.05 as thresholds for significantly differential methylation.

The differential expression analysis was performed using edgeR package in the R software, with |log2 Fold Change| > 1 and *p* < 0.05 as thresholds for significantly differential expression.

### Identification of candidate diagnostic methylation sites

The hypermethylated loci were mapped to corresponding genes based on the annotation of Illumina Human Methylation 450 arrays. Intersection of the downregulated genes and the genes that hypermethylated loci were mapped to were selected; then, hypermethylated loci that were not distributed within the promoter region were filtered out. The candidate diagnostic methylation sites were identified via an interaction between the methylation sites above and the hypomethylated sites in normal samples (*β* < 0.05).

### Assessment of diagnostic methylation sites

The logistic regression model for OSCC diagnosis was established based on the *β* values of the identified methylation sites in OSCC and paracancerous samples, using TCGA dataset as the training set. The specificity and sensitivity of this model was further evaluated using GEO datasets (GSE87053 and GSE123781) as the validation sets.

### Statistical analysis

The survival analysis was conducted using survival package in the R software, and then, Kaplan-Meier curve was plotted. Fisher’s exact test was adopted for calculation of the *p* value.

## Results

### DNA methylation overview and its effect on OSCC prognosis

**Fig. 1:**
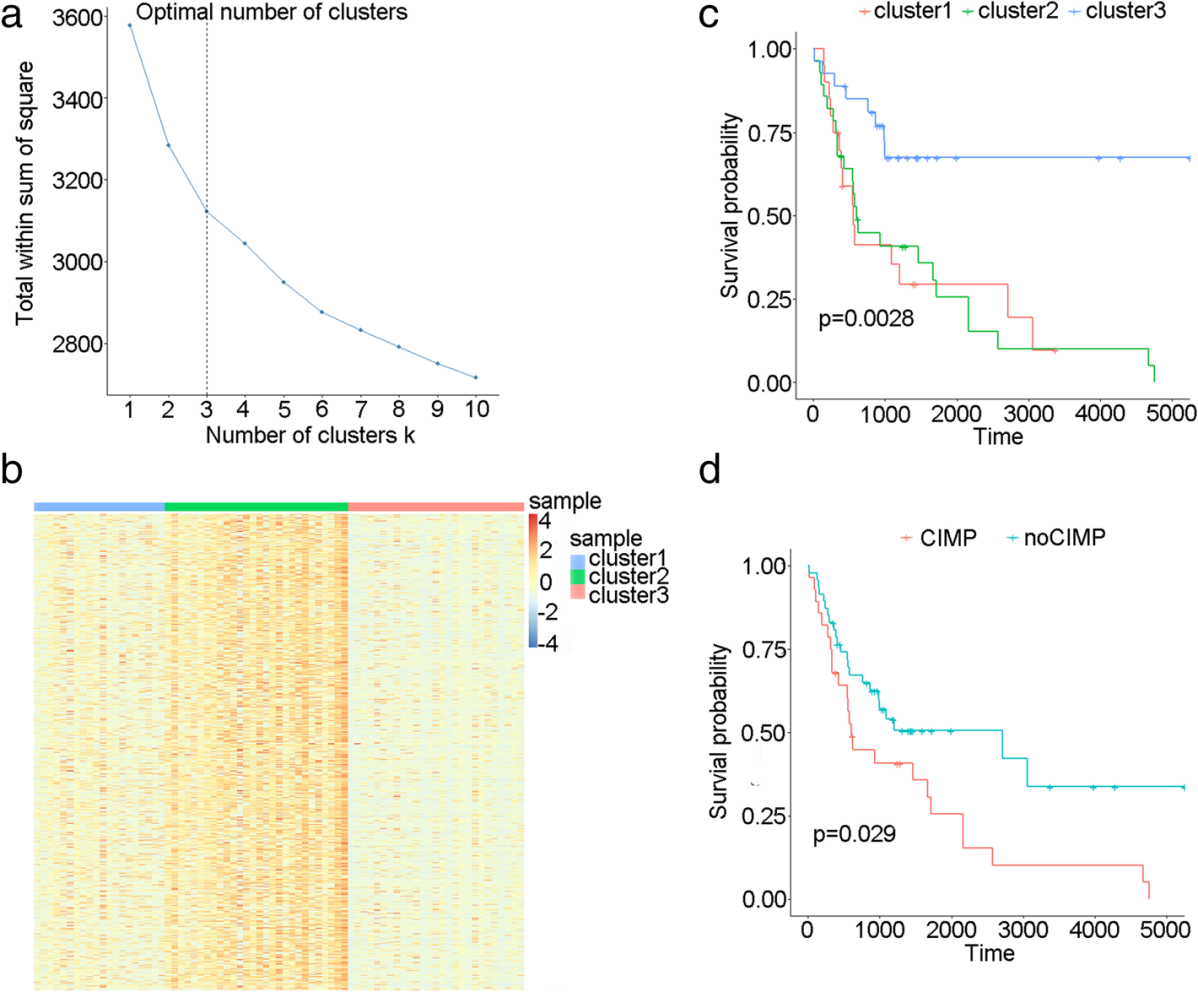
Compared with the OSCC samples without CIMP, the samples with CIMP presented lower survival probability. **a** We identified the optimal cluster number as 3 via within-cluster sum of squares method. **b** The heatmap of DNA methylation level of clusters 1, 2, and 3. **c** Survival curves of clusters 1, 2, and 3. **d** Survival curves of samples with/without CIMP

A total of 1243 methylation sites were selected with the criteria of SD > 0.2 in 70 OSCC patients and *β* < 0.05 in 5 paracancerous samples. The optimal cluster number was determined as 3 by using within-cluster sum of squares (wss) method (Fig. 1a). Then, the samples were clustered by using the Euclidean distance metric, and cluster 1, cluster 2, and cluster 3, which consisted of 20, 25, and 25 samples respectively, were identified. The heatmap of DNA methylation level showed that cluster 3 had the highest methylation level among these clusters (Fig. 1b); thus, we inferred that cluster 3 was probably characterized by CIMP.

Subsequently, survival analysis of the clusters was performed. As shown in Fig. 1c, cluster 2 had significantly decreased survival probability compared with clusters 1 and 3 (*p* = 0.0028). After merging of clusters 1 and 3, cluster 2 still presented lower survival probability (*p* = 0.029). The result indicated that the OSCC samples with CIMP had lower survival rate than those without CIMP. The 160 differentially expressed genes were screened in the group with good prognosis as compared with the group with poor prognosis (Figure S1), and 80 genes of them were upregulated and 80 were downregulated, as shown in Table S1.

### cg02860732 and cg04342955 were identified as candidate methylation sites for OSCC diagnosis

**Fig. 2:**
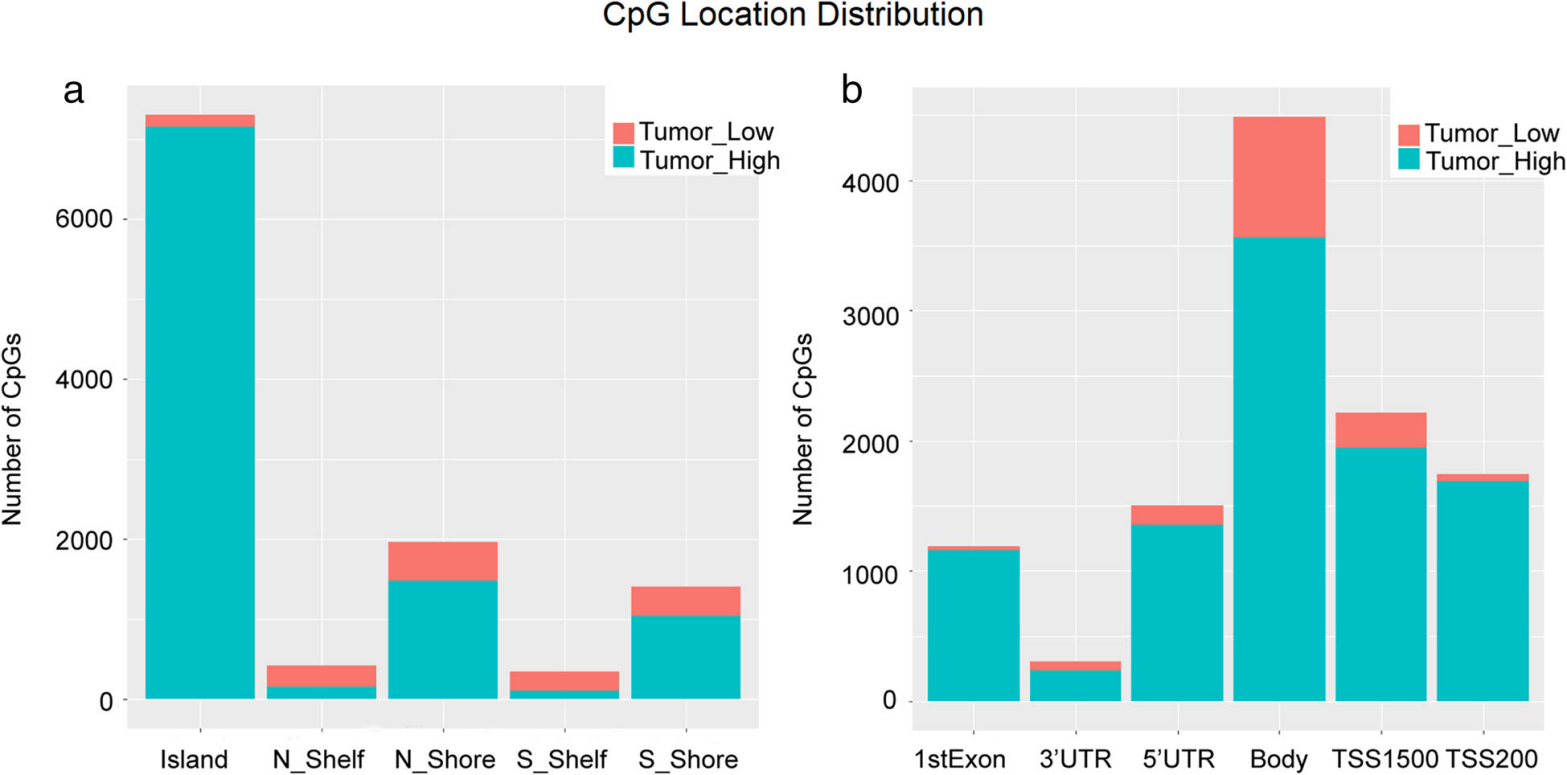
Distribution of the differentially methylated sites between the 5 paired OSCC and paracancerous samples. **a** Hypermethylated loci accounted for 96.7% of the methylation sites in CpG island. **b** Hypermethylated loci accounted for 92.3% of the methylation sites in gene promoter region (TSS200 and TSS1500)

**Fig. 3:**
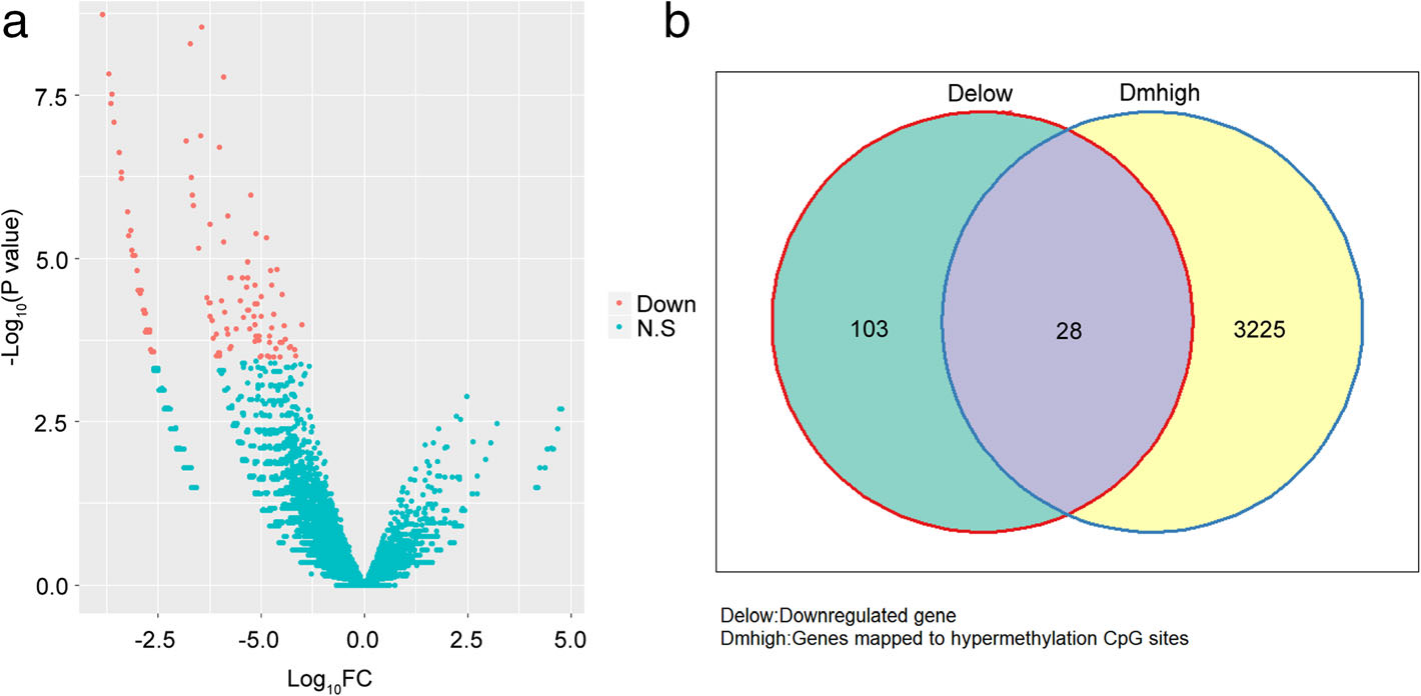
Differential methylation and expression analyses of OSCC samples. **a** Volcano plot of the differentially expressed genes between the 5 paired OSCC and paracancerous samples. **b** Shared genes between the genes that the hypermethylated loci were mapped to and the downregulated genes

A total of 23,315 differentially methylated sites between the 5 paired OSCC and paracancerous samples were obtained. As shown in Fig. 2, the hypermethylated loci were mainly distributed in CpG island (96.7%) and gene promoter region (TSS200 and TSS1500, 92.3%). These differentially methylated sites included 8090 hypomethylated loci and 15,225 hypermethylated loci, which corresponded to 2860 and 3253 genes respectively. There were 131 differentially expressed genes between the 5 paired OSCC and paracancerous samples, and all of them showed downregulated expressions (Fig. 3a). It is known that the hypermethylation of tumor suppressor gene, especially for the hypermethylation within the gene promoter region, could lead to the downregulation of the tumor suppressor gene and carcinogenesis [16, 17]. Therefore, interaction of the genes that hypermethylated loci were mapped to and the downregulated genes were obtained. As shown in Fig. 3b, there were 28 common genes between them, which contained 134 hypermethylated loci. After removal of 54 hypermethylated loci that were not located in the promoter region, an interaction of the remaining hypermethylated loci and the hypomethylated loci in normal samples (*β* < 0.05) was obtained. cg02860732 and cg04342955 were preliminarily screened as candidate diagnostic methylation sites for OSCC.

### Establishment and evaluation of the diagnostic model of OSCC

**Fig. 4:**
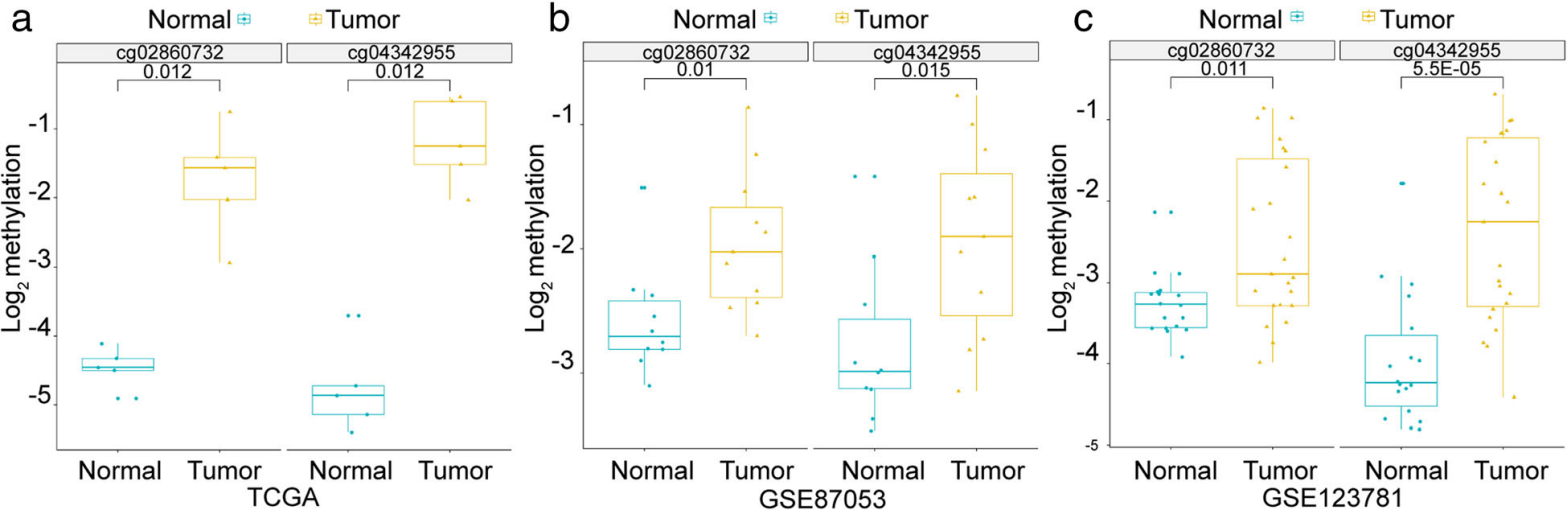
cg02860732 and cg04342955 had higher methylation level in OSCC tumor samples than that in normal controls in TCGA and GEO cohorts. **a** The methylation level of cg02860732 and cg04342955 in TCGA dataset. **b** The methylation level of cg02860732 and cg04342955 in dataset GSE87053. **c** The methylation level of cg02860732 and cg04342955 in dataset GSE123781

To validate the accuracy of cg02860732 and cg04342955, their methylation level in GEO datasets GSE87053 and GSE123781 were investigated. As shown in Fig. 4, both of the two methylation sites presented higher methylation level in OSCC tumor samples than that in normal controls in GEO datasets (*p* < 0.05), which was consistent with the tendency in TCGA dataset.

**Fig. 5:**
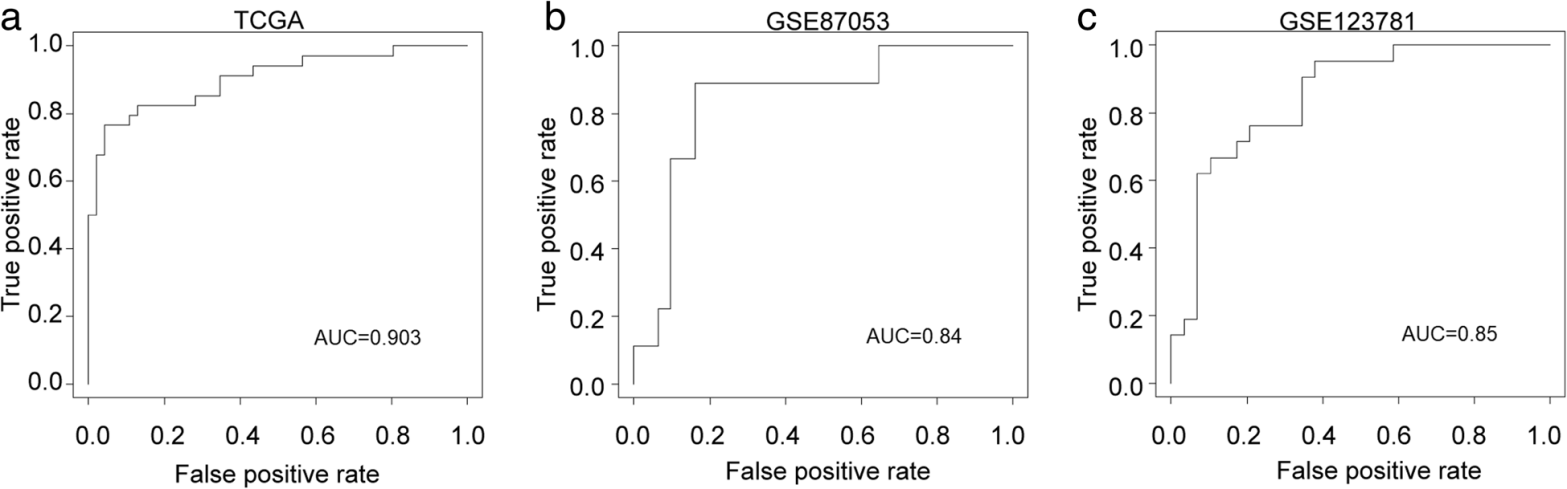
The logistic regression model presented relatively high diagnostic accuracy in OSCC. **a** The AUC for TCGA cohort. **b** The AUC for dataset GSE87053. **c** The AUC for dataset GSE123781

Then, the logistic regression model was established using the TCGA dataset as training set. As shown in Fig. 5a, the area under ROC curve (AUC) was 0.903 for the TCGA cohort. We validated the accuracy of this model in GEO datasets, achieving AUC of 0.84 for dataset GSE87053 and 0.85 for dataset GSE123781 (Fig. 5b and c). These results suggested that the logistic regression model we established with cg02860732 and cg04342955 presented relatively high diagnostic accuracy in OSCC.

## Discussion

Abnormal DNA methylation patterns, including hypermethylation of gene promoter region which is accompanied by gene silencing, play a key role in a large number of cancers [18]. Previous research revealed that the hub-methylated sites were related the diagnosis and prognosis for OSCC by weighted gene comethylation network analysis (WGCNA) [19]. To identify samples with CIMP, patients were clustered by K-means method in our study, and the results suggested that samples with CIMP had lower survival rate. Subsequently, a total of 131 downregulated genes and 23,315 differentially methylated sites consisting of 8090 hypomethylated loci and 15,225 hypermethylated loci between the OSCC tumor and paracancerous samples were identified. As the hypermethylation of tumor suppressor gene, especially hypermethylation within the gene promoter region, could lead to the downregulation of the tumor suppressor gene and carcinogenesis [16], we focused on the intersection of the genes that hypermethylated loci were mapped to and the downregulated genes here. Candidate diagnostic hypomethylated sites that were distributed within the promoter region of the 131 downregulated genes and presented hypomethylation in normal samples (β < 0.05) were further screened. Finally, methylation sites cg02860732 and cg04342955, locating in gene *SHISA9*, were preliminarily identified as diagnostic methylation sites for OSCC. Then, their methylation level in GEO datasets GSE87053 and GSE123781 were further investigated. In consistence with the tendency in TCGA cohort, cg02860732 and cg04342955 showed higher methylation level in OSCC tumor samples than that in normal controls in GEO datasets, validating the accuracy of the two methylation sites in different datasets.

Both of the methylation sites cg02860732 and cg04342955 were located in gene *SHISA9. SHISA9*, also known as *CKAMP44*, encodes type-I transmembrane protein which is postsynaptically localized and recognized as a member of Shisa protein family [20]. As an AMPA receptor-related protein, SHISA9 was observed in the complexes of AMPA receptor-related proteins and was able to control the short-term plasticity and currents modulated by AMPA receptor [21, 22]. Moreover, a previous study indicated that *SHISA9* overexpression resulted in more rapid and stronger desensitization of AMPA receptor, as well as weaker recovery from desensitization [23]. While AMPA receptors were reported to be involved in cancer development, such as proliferation, migration, and survival of cancer cells, and the AMPA receptor antagonists were considered as promising anticancer agents via inhibiting cancer cell viability [24]. It was found that AMPA receptor activation could enhance the migration and invasion of cancer cells in pancreatic cancer through Kras-MAPK signaling activation [25]. Besides, AMPA receptors were proved to promote the invasion and development of glioma [26]. Herein, we inferred that *SHISA9* might play an inhibitory role in OSCC by mediating the desensitization of AMPA receptor, which alleviated the activity of AMPA receptor [27]. As a consequence, the downregulated expression of *SHISA9* was probably associated with the OSCC carcinogenesis. However, the specific role of *SHISA9* in OSCC and the underlying mechanism warrant further investigation. To our knowledge, this was the first report of *SHISA9* as a potential tumor suppressor gene in OSCC.

In addition, a logistic regression model for OSCC diagnosis was identified based on cg02860732 and cg04342955, and obtained high AUC values for both of the training set and validation set. These results suggested that this logistic regression model presented relatively high diagnostic accuracy in OSCC. In clinical practice, early screening and accurate diagnosis of OSCC cases without obvious symptoms using conventional approaches remain to be difficult. Several studies have focused on DNA methylation analysis, attempting to explore methylation-related diagnostic biomarkers in OSCC [28, 29]. Compared with these researches that involved multiple genes, only gene *SHISA9* was implicated in the diagnostic model we identified, which was assumed to be more feasible.

In conclusion, our study identified cg02860732 and cg04342955 as potential diagnostic methylation sites for OSCC and a logistic regression model based on them for the diagnosis of OSCC which achieved good performance. These results provided a promising monitoring tool for OSCC diagnosis and might be helpful for timely treatment and therapy guidance of the patients.

## Supplementary Information

**Additional file 1.**

**Additional file 2: Table S1.** Analysis results of differentially expressed genes.

## Data Availability

The gene expression profiles of 75 OSCC samples were obtained from The Cancer Genome Atlas (TCGA, www.cancergenome.nih.gov).
