# Supplementary figures and images for: Identification of potential CpG sites for oral squamous cell carcinoma diagnosis via integrated analysis of DNA methylation and gene expression
Source: World J Surg Oncol. 2021 Jan 19;19:16. doi: 10.1186/s12957-021-02129-1 (PMC7816501; doi:10.1186/s12957-021-02129-1)

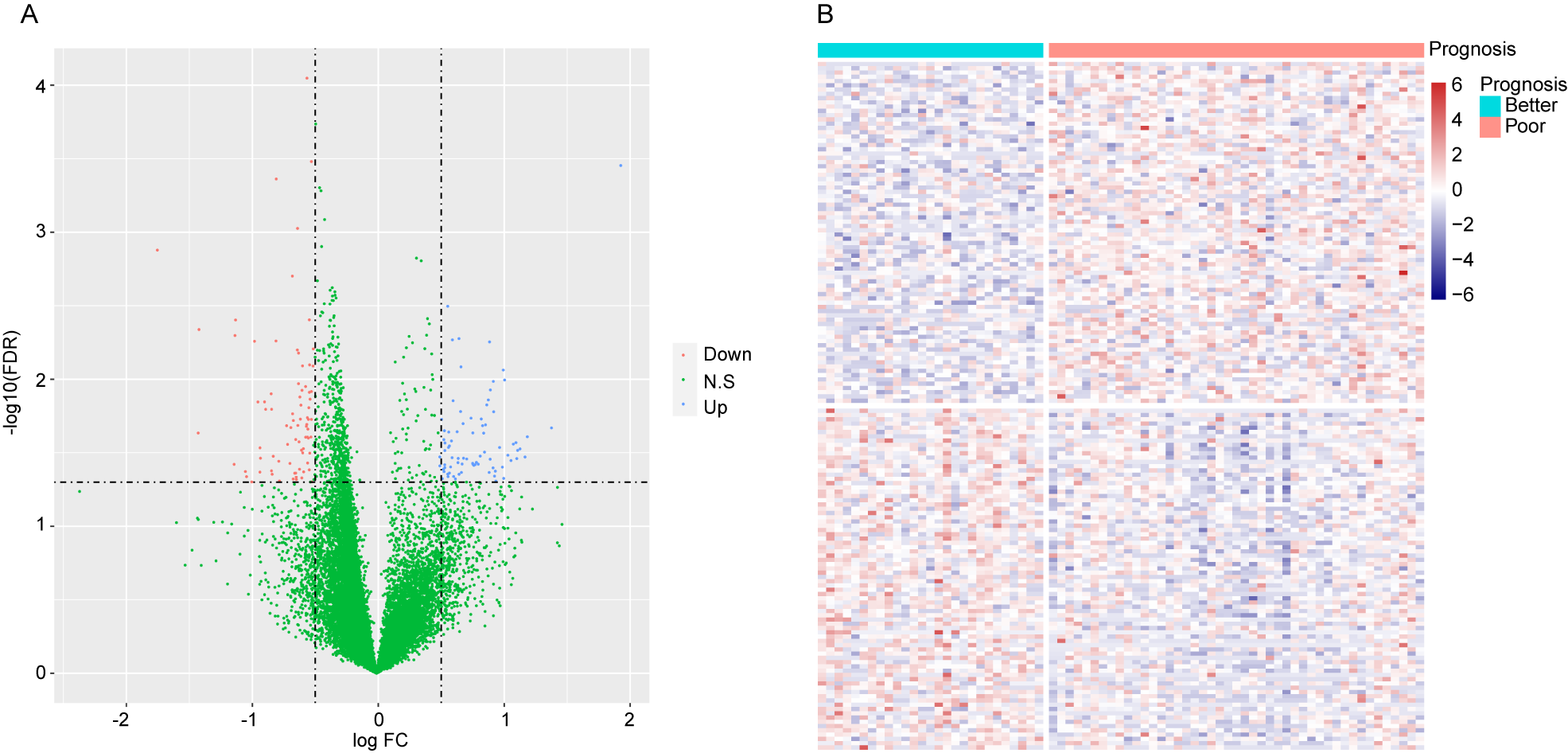

Supplement: Supplementary file 1 — Additional file 1. [file 12957_2021_2129_MOESM1_ESM.tif]
